
JOURNAL INFORMATION
==============================
NLM Title Abbreviation: Atlanta Med Surg J (1884)
NLM Journal ID: 101251623

Atlanta Medical and Surgical Journal (1884)

Publisher: Biomedical Journal Digitization Project

ARTICLE INFORMATION
==============================
PMCID: PMC9199288
PMID: 35829587
Article version: 1
Subjects: Original Communications

Hydrogen Peroxide in the Treatment of Puerperal Sepsis* Read before the Richmond Academy of Medicine and Surgery, October 11, 1898.

Upshur John N. M.D. Richmond, Va., Professor of the Practice of Medicine, Medical College of Virginia

Print publication date: 1898 Dec
Volume: 15
Volume Series: New Ser
Issue: 10
First page: 666
Last page: 668
License: This work is free of known copyright restrictions. For more information, please see PMC Back Issue Digitization.
License URL: https://creativecommons.org/publicdomain/mark/1.0/

==============================

liquor potassæ method, I have learned something to-night. By putting it on and rubbing it off he gets rid of that soapy product which is caused by the fatty secretion of the skin uniting with the liquor potassæ. That is a very valuable point. Now as to this peroxide of hydrogen; that is my own notion. I have seen unsatisfactory results from it, and I don't like to use it. About Dr. Childs's syphilitic vaccine sores; I have not seen any of them. The idea with me would be to follow the same principles, but I would use some form of mercury on these sores. My experience has been that it is best to use mercury locally on syphilitic sores. I agree with the gentlemen fully about ointment not being good for sores, but if you have an ointment that is sufficiently antiseptic, it will frequently do what the rubber tissue does, and you can use it on the face and elsewhere where you cannot use the rubber tissue, and it will do the work very well indeed. In treating these syphilitic sores, I would not hesitate to use ordinary mercurial ointment, either alone or with other things, and I think you will find that you will get a good effect from it.

*Dr. Childs:* I have used the white precipitate ointment on these sores with good results. I can't concur that all ointments are bad. There is not anything that gives better results in some cases than boracic acid ointment . I am a great believer in boracic acid ointment, and I use white precipitate ointment on these syphilitic sores.

---

### HYDROGEN PEROXIDE IN THE TREATMENT OF PUERPERAL SEPSIS.*

By JOHN N. UPSHUR, M.D., Richmond, Va.,

Professor of the Practice of Medicine, Medical College of Virginia.

Two principles of fundamental importance concerning puerperal sepsis are, first, that in these days of advanced asepsis, puerperal sepsis should not ordinarily occur, and, second, if it does occur, it should be treated aseptically rather than antiseptically. An exception to the first principle is found in such cases as are autogenetic

*Read before the Richmond Academy of Medicine and Surgery, October 11, 1898.

—a class of cases which, although their existence is denied by competent authority, the writer is convinced are sometimes encountered. These unpreventable ones are exemplified by instances of putrefaction and subsequent sepsis occurring in women in whose products of conception life has been extinct for several weeks.

When sepsis results from external causes, it is because the accoucheur or nurse has failed to secure surgical cleanliness. This in most instances is highly reprehensible. It is true that in the humble walks of life poverty, filth and ignorance are powerful factors in the causation of sepsis, and frequently triumph in spite of the physician's most watchful care. Elevation of temperature, not dependent upon some easily removable or transient causes, such as constipation or the first secretion of milk, but associated with scanty, offensive or absent lochia, is the invariable indication that infection has taken place, and that prompt clearing of the uterine cavity is imperative.

The writer's method of treatment in these cases is to first irrigate the interior of the uterus with a normal salt solution, remove secundines or other retained foreign materials by means of the sharp curette, then again irrigate freely with salt solution. After thoroughly drying with aseptic cotton or gauze, hydrogen peroxide is applied to the uterine cavity by means of a small intra-uterine syringe or an applicator upon which is wound a piece of aseptic gauze or absorbent cotton saturated with the agent. The foam should be removed and fresh applications made until the cessation of foaming gives positive evidence that the uterine cavity has been thoroughly cleansed. This procedure should be practiced daily until the temperature falls to normal and remains at that point. This, in the writer's experience, always occurs within a week. The following cases are illustrative of the efficacy of this mode of treatment:

CASE 1.—Mrs. H., aged 40, in her seventh labor, as the result of rigid cervix and violent uterine contractions, had rupture of the uterus in its long diameter, involving four-fifths of the thickness of the wall. Mural abscess and sepsis followed, associated with profuse, offensive lochia, the color of dirty dish-water. On the fifth day the uterus was above the pubis and spongy. The ordinarily recommended treatment was practiced without improvement, but on the eighth day the method above detailed, with hydrogen peroxide, etc., was instituted with the result that the temperature immediately fell to the normal point and the patient made a good recovery.

CASE 2.—Mrs. D., delivered of her third child two months prematurely. Baby, much emaciated in consequence of interference with nutrition from placental degeneration, lived twelve hours. Within the first five days the temperature ranged from 101° to 105° Fahr., and the usual concomitant symptoms of sepsis were present. On the sixth day after delivery, curettage with free douching of hot salt solution was practiced and the usual application of hydrogen peroxide was made. Temperature taken half hour after treatment showed a fall of one degree, while on the seventh day it was normal. From this date on convalescence was uninterrupted and the patient was out of bed as early as though no complication had occurred.

CASE 3.—Mrs. S., after rapid delivery, did well for nine days, when the usual symptoms of puerperal sepsis appeared, due in all probability to her wretched surroundings, lack of proper nursing, etc. The treatment above detailed was exhibited, the temperature promptly returned to normal, and there was speedy and satisfactory convalescence.

The *rationale* of the treatment by hydrogen peroxide is that this agent causes a rapid oxidation or superoxidation of effete organic matter, thus completing in a very short time what it would take the unassisted process of nature a dangerously long period to accomplish. It initiates, but infinitely improves and accelerates the efforts of the human organism to remove offending foreign materials. The advantage of this agent over mercuric chloride, carbolic acid, and other agents that act chemically, is that it is non-corrosive and non-destructive of healthy tissue. Furthermore, the results obtained from the use of hydrogen peroxide are vastly superior to those obtained by the use of any other agent, so that the writer now approaches the treatment of puerperal sepsis with less fear of unfortunate results than he has ever before experienced.

*210 West Grace Street.*
